# Evidence of Bacterial Biofilms among Infected and Hypertrophied Tonsils in Correlation with the Microbiology, Histopathology, and Clinical Symptoms of Tonsillar Diseases

**DOI:** 10.1155/2013/408238

**Published:** 2013-12-24

**Authors:** Saad Musbah Alasil, Rahmat Omar, Salmah Ismail, Mohd Yasim Yusof, Ghulam N. Dhabaan, Mahmood Ameen Abdulla

**Affiliations:** ^1^Department of Microbiology, Faculty of Medicine, MAHSA University, 59100 Kuala Lumpur, Malaysia; ^2^Pantai Hospital Cheras, 56100 Kuala Lumpur, Malaysia; ^3^Institute of Biological Science, Faculty of Science, University of Malaya, 50603 Kuala Lumpur, Malaysia; ^4^Department of Medical Microbiology, Faculty of Medicine, University of Malaya, 50603 Kuala Lumpur, Malaysia; ^5^Department of Biomedical Science, Faculty of Medicine, University of Malaya, 50603 Kuala Lumpur, Malaysia

## Abstract

Diseases of the tonsils are becoming more resistant to antibiotics due to the persistence of bacteria through the formation of biofilms. Therefore, understanding the microbiology and pathophysiology of such diseases represent an important step in the management of biofilm-related infections. We have isolated the microorganisms, evaluated their antimicrobial susceptibility, and detected the presence of bacterial biofilms in tonsillar specimens in correlation with the clinical manifestations of tonsillar diseases. Therefore, a total of 140 palatine tonsils were collected from 70 patients undergoing tonsillectomy at University Malaya Medical Centre. The most recovered isolate was *Staphylococcus aureus* (39.65%) followed by *Haemophilus influenzae* (18.53%). There was high susceptibility against all selected antibiotics except for cotrimoxazole. Bacterial biofilms were detected in 60% of patients and a significant percentage of patients demonstrated infection manifestation rather than obstruction. In addition, an association between clinical symptoms like snore, apnea, nasal obstruction, and tonsillar hypertrophy was found to be related to the microbiology of tonsils particularly to the presence of biofilms. In conclusion, evidence of biofilms in tonsils in correlation with the demonstrated clinical symptoms explains the recalcitrant nature of tonsillar diseases and highlights the importance of biofilm's early detection and prevention towards better therapeutic management of biofilm-related infections.

## 1. Introduction

The ear, nose, and throat (ENT) represent a natural habitat for a broad range of microorganisms such as commensal bacteria as well as potential pathogens [1]. However, these bacteria can sometimes find their way to overcome the defense barriers of such locations and establish chronic infections that poses a challenge to both medical practice and healthcare system [2]. Infections of the ENT such as tonsillitis are diseases that occur with high frequency [3]. During the past decades, efforts have been made to manage the infectious diseases of tonsils [4]. It has been reported that the impact of tonsillar diseases may not only affect the tonsils alone but it can reach other related anatomic structures like the paranasal sinus, upper aerodigestive tract, and Eustachian tube-middle ear complex [4]. Thus understanding the microbiology and pathophysiology of such diseases represents an important step in the management of biofilm-related infections.

Chronic infections of the ear, nose, and throat are becoming more resistant to common antimicrobial therapies [5] due to the ability of bacteria to persist through the formation of biofilms [6] which are bacterial cells attached to a surface and embedded in a matrix of exopolysaccharide [7]. The most important step in biofilm formation is the secretion of a matrix comprising of proteins and sugars outside the individual bacterial cells [8]. In addition, the biofilm structure provides mechanical stability to the bacteria and it represents a site where genetic elements are exchanged [9]. It has been estimated that more than 65% of all human bacterial infections are associated with biofilms [10]. Moreover, bacteria in the biofilm are 1000 times more resistant to antibiotics than their free-living counterparts [11, 12] which may lead to discrepancies between the *in vitro* and *in vivo* antimicrobial susceptibility results [13]. Therefore, shifting the mode of antibiotic regimens to include bacteria in a biofilm mode will improve the methods of treatment especially against biofilm-associated infections [14]. Biofilms play a major role in chronic tonsillitis which is considered one of the most common pathologies in childhood [15–17]. Despite the widespread use of antibiotics, tonsillitis is often recalcitrant and tonsillectomy is mainly performed only when antibiotic therapy fails to relieve the symptoms of infection [18] or when the enlarged tonsils cause functional obstruction to the air passage [19]. Moreover, the increasing incidence of *β*-lactamase-producing bacteria recovered from tonsils may protect the causing pathogens from being eliminated by host defense and antibiotics [20] which may lead to the recurrence of tonsillar infections that are caused by microorganisms shown to be susceptible *in vitro* [21]. These observations have led to the hypothesis that bacteria in a biofilm can resist eradication causing chronic inflammation and permanent changes in the tonsillar lymphoid tissue [21].

A biofilm is considered a marker of virulence which can be detected phenotypically [22]. However, proper visualization of biofilms within tissue sections is challenging due to the difficulty in staining both bacteria and glycocalyx [6]. Most of the early investigations on biofilms relied heavily on scanning electron microscopy (SEM) [23]. It has been reported that the most effective and nondestructive approach for examining biofilms within tissue sections is via confocal laser scanning microscopy (CLSM) [24]. In our study, we have identified the bacterial isolates recovered from tonsillar specimens and evaluate their antimicrobial susceptibility in addition to examining the histopathology and presence of bacterial biofilms in tonsillar tissue sections in correlation with the clinical manifestations of tonsillar diseases that are due to infection and obstruction.

## 2. Materials and Methods

### 2.1. Selection of Patients

A total of 70 patients undergoing elective tonsillectomy were enrolled in this study. Patients were diagnosed with three main clinical cases including recurrent tonsillitis, chronic tonsillitis, and obstructive sleep apnea. The duration of the study was 10 months from October 2009 to July 2010. Prior to surgery, an approval letter was obtained from the medical ethics committee at University Malaya Medical Centre (UMMC) PPUM/UPP/300/02/02 Ref. number 744.11 and written consents were recorded from each patient separately. Inclusion criteria included 3 attacks/year of chronic and recurrent tonsillitis or 5 attacks in 2 years with symptoms like fever, snoring, sore throat, and inability to take normal diet [25]. Other inclusion criteria included patients diagnosed with obstructive sleep apnea with symptoms like nocturnal snoring with partial upper airway obstruction, complete cessation of airflow with gas exchange abnormalities, and severe disturbance of sleep [26]. Exclusion criteria included patients with a history of infection who received antimicrobial therapy within one month prior to surgery, patients with grossly asymmetrical tonsillar size as noted on preoperative clinical assessment, patients undergoing tonsillectomy for emergency conditions such as peritonsillar abscess or other deep neck space infections, and patients suspected for benign or malignant tonsillar tumors [27]. Other exclusion criteria included immunocompromised and diabetic patients [28] and patients with obstructive sleep apnea that are not due to adenotonsillar hypertrophy but to other causes such as craniofacial anomalies and neurologic abnormalities [29].

### 2.2. Indications of Tonsillar Diseases

The clinical indications for tonsillectomy were used as a guideline to determine the assignments of tonsillar diseases among the selected patients [27]. The size of tonsils was estimated on a 1+ to 4+ scale as outlined in the group classification [30] and the grading of tonsillar hypertrophy [31]. Patients were classified into two main groups based on their clinical diagnosis and history of infection; the first group was designed the name tonsillar infection group represented by 49 patients with recurrent tonsillitis having minimally visible tonsils occupying less than 25% of the oropharyngeal airway (1+) and 9 patients with chronic tonsillitis having moderately enlarged tonsils occupying less than 50% of the oropharyngeal airway (2+). The second group was designed the name tonsillar obstruction group represented by 12 patients with obstruction sleep apnea having moderately to massively enlarged tonsils (3+ or 4+) occupying greater than 50–75% of the oropharyngeal airway.

### 2.3. Collection of Tonsillar Specimens

Upon surgery, the surface of palatine tonsils was swabbed with a sterile cotton applicator followed by the surgical removal [28]. Tonsillar biopsies were aseptically dissected into four parts [32]; the first part was unfixed and was referred to the Clinical Diagnostic Laboratory (CDL) at UMMC along with the tonsillar swabs to identify the type of microorganisms. The second part was fixed with 4% glutaraldehyde to detect the presence of biofilms via SEM. The third and fourth parts were fixed with 10% neutral buffered formalin to detect the presence of biofilms via CLSM and examine the histopathology of tonsils respectively.

### 2.4. Isolation of Tonsillar Microorganisms

Tonsillar biopsies were aseptically weighted and placed in thioglycollate broth with a volume equivalent to 1 : 10 dilution followed by tissue homogenization. Serial dilutions of 1 : 10 and 1 : 100 were performed and each dilution was poured into Columbia agar supplemented with 5% sheep blood. After incubation for 24 hours, the microbial load was assessed by colony counting as described previously [33] and bacterial identification was accomplished by routine culturing on selective and differential media. Moreover, biochemical tests were performed such as DNase test for *S. aureus*; optochin test, bile solubility test, and bacitracin test for Streptococci spp.; indole, citrate test, malonate utilization test, urease test, oxidase test, and methyl red test for *Enterobacteriacea*; and the XV factor test for *Haemophilus *spp.

### 2.5. Antimicrobial Susceptibility of Tonsillar Isolates

The Clinical Diagnostic Laboratory in consultation with the infectious disease practitioners at UMMC has decided which antimicrobial agent to report routinely or selectively. Therefore, susceptibility test for selected antibiotics was carried out via disk diffusion as descripted previously [34]. Briefly, a fixed volume of nutrient broth containing a standard concentration for each bacterial isolate was smeared evenly onto the surface of Mueller-Hinton agar plate and filter paper disks impregnated with antibiotic concentrations were applied to the plate surface followed by aerobic incubation. The zone of inhibition for each antibiotic was measured and the edge of these zones correlated with the antibiotic concentration that inhibits the growth of bacteria were compared to a standard table of predetermined zone widths [35].

### 2.6. Histopathology Examination of Tonsils

Tonsillar biopsy specimens were examined by routine staining with hematoxylin & eosin (H&E) as described previously [36]. Briefly, biopsies were embedded in paraffin wax then cut using a manual rotary microtome (Leica RM2235, Leica Microsystems. Germany) into thin sections that were later fixed onto a glass slide. Slides were then deparaffinized in xylene for 10 minutes and then rehydrated for 1 minute in a grade series of ethanol. Sections were then stained with hematoxylin for 2 minutes, rinsed, and then stained with eosin for 1 minute. Slides were then dehydrated with ethanol followed by xylene for 10 minutes and mounted to be inspected under light microscope.

### 2.7. Microscopic Examination of Biofilms

To visualize the biofilm presence covering the surface of tonsils, SEM was used as described previously [6]. Briefly, specimens were fixed in 4% glutaraldehyde for 24 hours followed by dehydration through a graded series of acetone solutions and then critical point drying was performed for which they were mounted on metal stubs and coated with gold prior to imaging. Specimens were examined by SEM (INCA x-sight, Oxford instruments. UK). Images were collected at an acceleration voltage of approximately 5.0 kV, a filament current of approximately 10^−10^ A, and a working distance of approximately 39 mm; images were digitized as high resolution TIFF files and were then converted to high-quality TIF files using commercially available software. To visualize the biofilm's 3D architecture, CLSM was used in combination with immunohistochemistry staining for which a fluorescent-labeled lectin named concanavalin A (Con A) will specifically bind to the biofilm's matrix as described previously [6]. Briefly, specimens were embedded in an optimal cutting temperature (OCT) media and were frozen in a mixture of cold isopentane and liquid nitrogen forming blocks that were cut into a thickness of 5–10 *μ*m using a cryostat (Leica CM1850, Leica Microsystems. Germany), then fixed onto a glass slide. Staining was achieved with Propidium iodide followed by Con A and sections were then embedded in an antiquenching mounting medium of phosphate-buffered saline and glycerol. Specimens were examined by CLSM (LSM 700. Carl Zeiss. Germany) and various colocalization parameters were determined with the aid of ZEN 2010 software for a more comparative analysis of biofilms. Specimens were considered having a biofilm if more than one biofilm structure was observed at the surface or within the crypts of tonsils. However, when only bacteria were visualized without any matrix surrounding them they were not considered having a biofilm [9].

## 3. Results

### 3.1. Prevalence of Clinical Cases

The prevalence of clinical cases in tonsillar infection group was 20 (28.57%) cases of recurrent tonsillitis among paediatric patients and 29 (41.42%) cases among adult patients, whereas 4 (5.71%) cases of chronic tonsillitis among paediatric patients and 5 (7.14%) cases among adult patients. Moreover, the prevalence of clinical cases in tonsillar obstructive group was 9 (12.85%) cases of obstructive sleep apnea among paediatric patients and 3 (4.28%) cases among adult patients. In recurrent tonsillitis, the age group of 1.0–10 years old was the highest with 18 (25.71%) patients followed by the 11–20 years with 16 (22.85%) patients and the 21–30 years with 14 (20%), whereas 31–40 years and 41–50 years were among the lowest with 3 (4.28%) and 1 (1.42%) patients, respectively. In chronic tonsillitis cases, the age group of 11–20 years old was the highest with 5 (7.14%) patients followed by the 1–10 years with 3 (4.28%) patients and the 21–30 years with 1 (1.42%) patient. Moreover, the highest number of age group in obstructive sleep apnea cases was the 1.0–10 years old with 7 (10%) patients followed by 11.0–20 years with 2 (2.85%) patients. The frequency and type of operative procedures performed on selected patients showed that, among all clinical cases, 44 (62.85%) patients underwent tonsillectomy alone while 26 (37.14%) patients underwent tonsillectomy and adenoidectomy (T&A). Our results showed that the clinical symptoms were correlated with the presence of biofilms in the tonsils (Table 1). A significantly higher percentage of patients presented chronic or recurrent infections rather than obstruction manifestation (*P* < 0.05). However, an association between the clinical symptoms like snore, apnea, nasal obstruction, and tonsillar hypertrophy were found to be related to the presence of bacterial biofilms in the tonsils.

### 3.2. Microbiology of Tonsillar Diseases

The weight of excised tonsils varied from 2.2 to 8.1 grams. There was no correlation between the tonsillar weight and the number and type of bacterial isolates. In addition, there was no significant difference in the recovery rate of isolates among the clinical cases. Recurrent tonsillitis cases showed a recovery average of 10.85 isolates/gram tonsil and chronic tonsillitis cases showed 3.75 isolates/gram tonsil whereas obstructive sleep apnea cases showed 3 isolates/gram tonsil. The total number of bacterial isolates recovered from tonsillar specimens was 464 isolates with 184 (39.65%) isolates of *Staphylococcus aureus* as the most common followed by 86 (18.53%) isolates of *Haemophilus influenzae* and 56 (12.06%) isolates of* Streptococcus agalactiae*. There was no significant difference between the number of isolates recovered from both tonsillar swab and biopsy specimens. However, isolates of *Haemophilus parainfluenzae* were more frequently recovered in the core of tonsils 10 (2.15%) rather than the surface 21 (4.52%). Distribution of bacterial isolates among tonsillar specimens is shown in (Table 2). Moreover, a special group of pathogens designated the name ESKAPE was isolated from tonsillar specimens; these were including *Enterococcus faecium*, *Staphylococcus aureus*, *Klebsiella pneumoniae*, *Acinetobacter baumannii*, *Pseudomonas aeruginosa*, and *Enterobacter* species. The total number of recovered ESKAPE pathogens was 225 isolates (48.46%) with 184 isolates (39.65%) of *Staphylococcus aureus*, 30 isolates (6.46%) of *Klebsiella pneumoniae*, 9 isolates (1.93%) of *Pseudomonas aeruginosa,* and 1 (0.21%) isolate for each of *Acinetobacter baumannii* and *Enterobacter cloacae* with no *Enterococcus faecium* isolates.

### 3.3. Antimicrobial Susceptibility of Tonsillar Diseases

**Figure 1:**
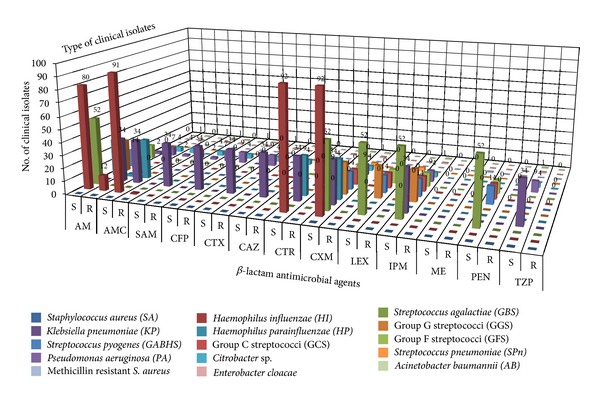
Antimicrobial susceptibility of tonsillar isolates against selected *β*-lactam agents. AM: ampicillin, AMC: amoxicillin-Clavulanic acid, SAM: ampicillin-Sulbactam, CFP: cefoperazone, CTX: cefotaxime, CAZ: ceftazidime, CTR: ceftriaxone, CXM: cefuroxime, LEX: cephalexin, IPM: imipenem, ME: methicillin, PEN: penicillin, TZP: piperacillin-Tazobactam. (S) indicates susceptible isolates and (R) indicates resistant isolates.

**Figure 2:**
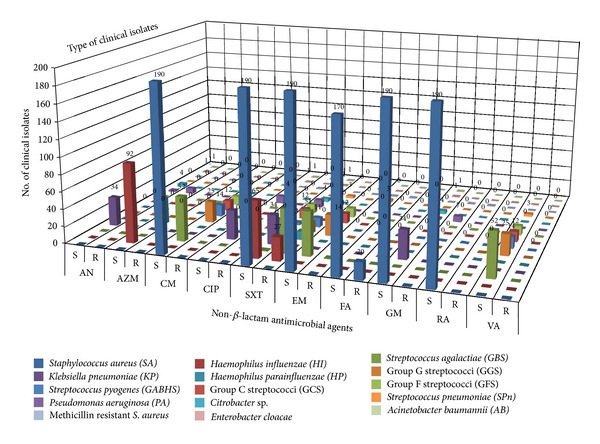
Antimicrobial susceptibility of tonsillar isolates against selected non-*β*-lactam agents. AN: amikacin, AZM: azithromycin, CM: clindamycin, CIP: ciprofloxacin, SXT: co-trimoxazole, EM: erythromycin, FA: fusidic Acid, GM: gentamicin, RA: rifampin, VA: vancomycin. (S) indicates susceptible isolates and (R) indicates resistant isolates.

The results of antimicrobial susceptibility of *S. aureus* isolates showed that 169 (91.48%) isolates were susceptible to all the selected antibiotics whereas 20 (10.87%) isolates were resistant to fusidic acid and only 1 (0.5%) isolate was resistant to both methicillin and fusidic acid [37]. The antibiotic cotrimoxazole showed the highest rate of resistance against majority of the bacterial isolates including Group A beta haemolytic streptococci (GABHS) with 11 (2.37%) resistant and 3 (0.64%) susceptible; Group B *streptococcus* with 55 (11.85%) resistant and 1 (0.21%) susceptible; Group G Streptococci with 14 (3.01%) resistant and 11 (2.37%) susceptible; *Streptococcus pneumoniae* with 3 (0.64%) resistant; *Haemophilus influenzae* with 27 (5.81%) resistant and 59 (12.71%) susceptible, and *Haemophilus parainfluenzae* with 10 (2.15%) resistant and 21 (4.52%) susceptible. The number of *Haemophilus influenzae* isolates that were *β*-lactamase negative ampicillin-resistant (BLNAR) was 12 (2.58%) isolates. Resistance to antimicrobial agents belonging to the penicillins class was detected including 3 (0.64%) isolates of *Streptococcus pneumoniae* resistant to penicillin, 12 (2.58%) isolates of *Haemophilus influenzae* resistant to ampicillin, 30 (6.46%) isolates of *Klebsiella pneumoniae* resistant to ampicillin, 7 (1.50%) isolates of *Pseudomonas aeruginosa* resistant to ampicillin, 4 (0.86%) isolates of *Citrobacter* sp. resistant to ampicillin, and 1 (0.21%) isolate of each of *Acinetobacter baumannii,* and *Enterobacter cloacae* resistant to ampicillin. A total of 10 (2.15%) isolates recovered from infected tonsils were multidrug resistant (MDR) whereas 7 (1.50%) isolates recovered from hypertrophied tonsils were MDR including 7 isolates of *Pseudomonas aeruginosa*, 3 isolates of *Streptococcus pneumoniae *and 1 isolate of *Enterobacter cloacae*. There were an increased number of *H. influenzae* isolates in association with GABHS which may be due to a synergistic relationship between these organisms. The antimicrobial susceptibility of tonsillar bacterial isolates against selected *β*-lactam and non-*β*-lactam agents is shown in (Figures 1 and 2) respectively.

### 3.4. Histopathology of Tonsillar Diseases

The gross pathology examination of tonsillar specimens showed the excised palatine tonsils as a nodular to tubular irregular brownish and soft surface tissue with an average measuring size of 2 × 2 × 1 cm. However, the microscopy examination revealed that the tonsillar tissue is covered with benign stratified squamous epithelium with the stroma consisting of variably sized reactive lymphoid follicles. No malignancies were found and a rate of crypt keratination was observed in majority of the tissue sections. The overall pathological interpretation was described as reactive (benign) lymphoid hyperplasia. Moreover, there was evidence of infection with *Actinomyces* spp. in 11 (15.71%) tonsillar biopsies. These infections caused an inflammatory lesion of the tonsillar crypts and led to tonsillar hypertrophy. The most frequent rate of tonsillar grading was grade III (3+) with 39 (55.71%) patients followed by grade II (2+) with 20 (28.57%) patients and grade I (1+) with 6 (8.57%) and patients then grade IV (4+) with 5 (7.15%) patients. Tonsillar biopsies from patients with chronic and recurrent tonsillitis showed increased number of lymphatic follicles in comparison to patients with obstructive sleep apnea. Moreover, an association with adenoids hypertrophy was detected in 22 (32.85%) patients.

### 3.5. Evidence of Bacterial Biofilms

**Figure 3:**
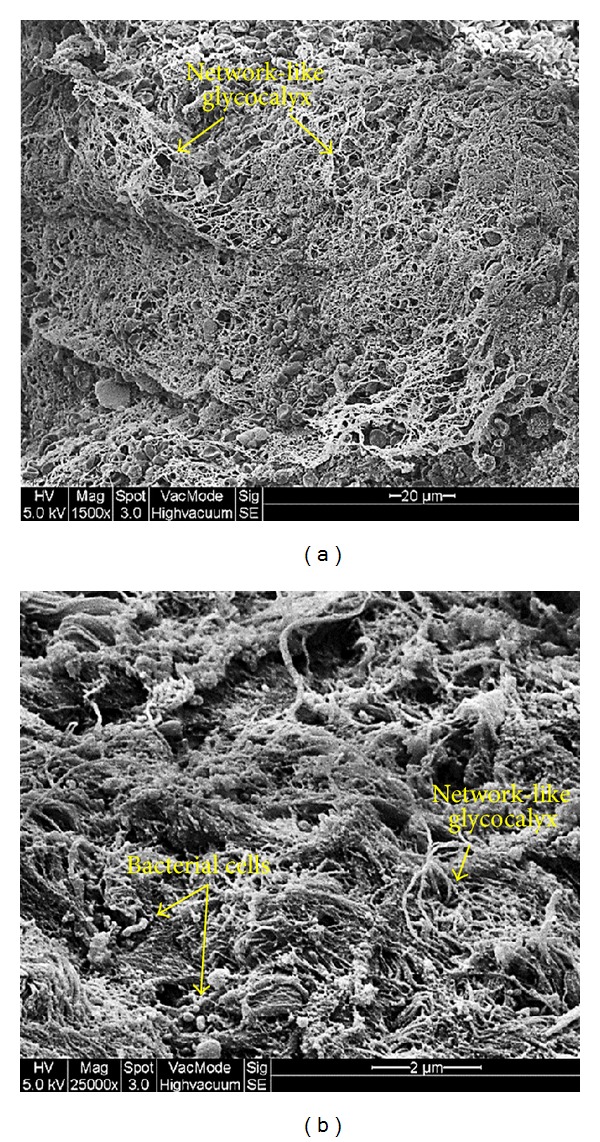
Microscopic evidence of bacterial biofilms on the tonsillar surface via SEM. (a) Overall image of biofilm from a patient with recurrent tonsillitis showing the layers of network-like glycocalyx (low magnification 1500x). (b) Representative image of biofilm from a patient with chronic tonsillitis showing bacterial cells attached to the surface of tonsillar cells and embedded in a network-like glycocalyx (high magnification 25000x). Arrows indicate the biofilm structures.

**Figure 4:**
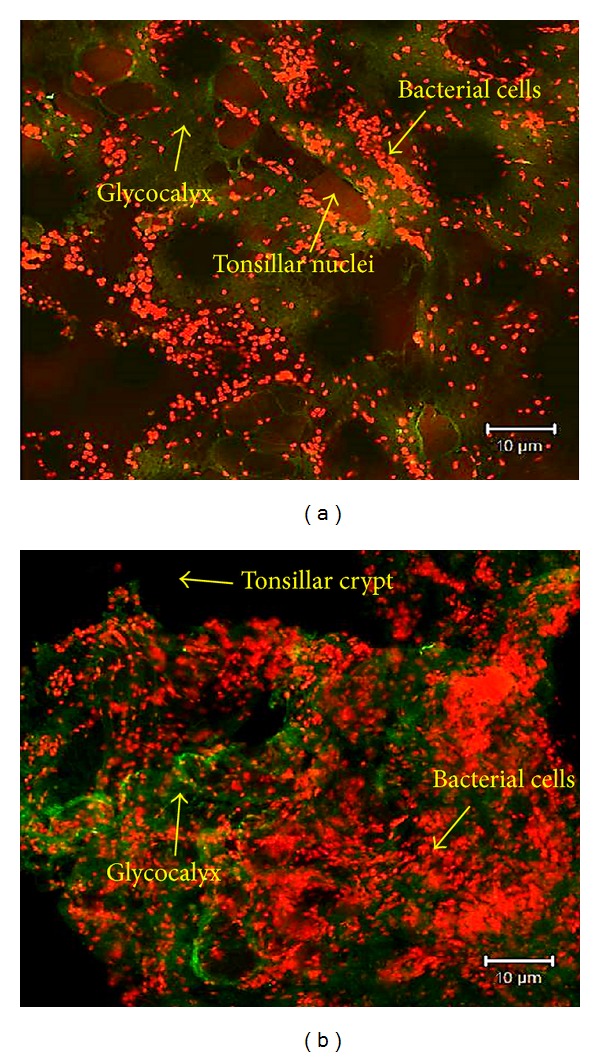
Microscopic evidence of bacterial biofilms within the tonsillar crypts via CLSM. (a) Representative image of biofilm from a patient with obstructive sleep apnea showing bacterial cells (red) embedded with glycocalyx (green) surrounding the tonsillar nuclei (red). (b) Three-dimensional image of a biofilm showing bacterial aggregates (cells) embedded in a glycocalyx matrix (100x). Arrows indicate the biofilm structures and tissue sections were stained with propidium iodide and concanavalin A.

Microscopic examination of biofilms in the tonsils via SEM showed abnormal tonsillar mucosal surrounded by red blood cells along with small depressions between the epithelium harboring bacterial microcolonies and some inflammatory cells at the periphery. Attached bacteria were present on the surface and were clearly distinguished from smaller irregularities nearby. Bacterial cells seemed to be organized in a scaffolding network and were connected by an extracellular matrix (Figure 3). Examination of the biofilm's 3D structure via CLSM showed evidence of accumulated bacteria embedded in an amorphous polysaccharide matrix that underlines the tonsillar crypts (Figure 4). Evidence of biofilms were present in 30 out of 49 patients with recurrent tonsillitis, 5 out of 9 patients with chronic tonsillitis, and 7 out of 12 patients with obstructive sleep apnea. Double staining showed that bacterial cells and tonsillar cells were stained red whereas the biofilm's glycocalyx was stained fluorescent green. Majority of the visualized bacteria were cocci shaped with some bacilli indicating a polymicrobial biofilm community. However, the type of bacteria could not be identified based on the microscopic examination. Colocalization analysis showed red tonsillar nuclei tagged with propidium iodide and green glycocalyx tagged with Con A.

## 4. Discussion

Our assessment for the microbiology of tonsillar diseases showed that *Staphylococcus aureus* was the most common bacterial isolate followed by *Haemophilus influenzae* which indicates that those two pathogens might be the etiological factors for chronic and recurrent tonsillitis. This was similar to Kielmovitch et al. in which they have reported *S. aureus* and *H. influenzae* as the main causative agents of tonsillitis [38]. There was low number of recovery among *Streptococcus pneumoniae* and GABHS isolates from both infected and hypertrophied tonsils which indicates their less possible role in the development of chronic and recurrent tonsillitis in addition to obstructive sleep apnea. This was in contrast with Kielmovitch et al. where they have reported GABHS, *Streptococcus pneumonia,* and *Neisseria gonorrhoeae* as the main causes of tonsillitis [38].

In our study, there was no significant difference between the tonsillar surface and core. In fact, the same type of bacteria that were isolated from the core was isolated from the surface as well. These findings were similar to those of Almadori et al. where they have reported no qualitative difference between tonsillar surface and core cultures [39]. However, this was in contrast with Brook et al. and Rosen et al. were they have reported that the isolated microorganisms from tonsillar surface may not always represent the real cause of recurrent tonsillitis [40, 41]. The only microorganism that was found to have significant difference in the recovery was *Haemophilus influenzae* for which 10 isolates where recovered from the core whereas 21 were recovered from the surface. This was similar to Gul et al. where they have reported a difference in recovery between surface and core tissue among *H. influenzae* and *S. aureus* isolates [42]. *H. influenzae* was rarely recovered from the tonsillar surface which indicates that the surface cultures commonly show normal flora whereas the tonsil core cultures show pathogenic microorganisms. Despite the contrast with previous studies in the role of swabbing, the use of swabs can still be reliable to recognize the presence of possible pathogens especially for patients who are not willing to undergo surgical management despite of not being responding to antimicrobial treatment.

In the case of tonsillar infection, bacteria that inhabit the crypts can spread into the tissue and secret their toxins leading to infiltration of leukocyte and surface ulceration that can cause the bacteria to inoculate the tonsillar core [43, 44]. However, the mechanism of activating such infections is still poorly understood [28]. Therefore, knowing the microbiology of tonsils does not help in the treatment of disease however; it establishes an understanding whether the bacteria play a role in reactivating recurrent infections by using virulence factors such as forming a biofilm.

Our antimicrobial susceptibility results showed a high rate of sensitivity among majority of tonsillar isolates. This was similar to Sadoh et al. in which they have reported a 100% sensitivity to cefuroxime, azithromycin, and ceftazidime among *S. aureus* and *β*-haemolytic streptococci [45]. It is worthy of note that ampicillin exhibited more resistance against pathogens such as *P. aeruginosa* and *H. influenzae*. Although the reason for this difference is not clear, we suspect it may be related to possible abuse of the easily accessible and relatively cheap ampicillin that will eventually develop resistance. Moreover, a noticeable percentage of resistance to the antibiotic cotrimoxazole was detected; these include 21.43% resistance by GABHS isolates, 68.60% by *H. influenzae* isolates, and 67.75% by *H. parainfluenzae* isolates. This was similar to Sadoh et al. where they have reported no sensitivity to ampicillin and cotrimoxazole [45]. Although our susceptibility results cannot estimate the current status of antimicrobial resistance in Malaysia, it highlights a number of important issues regarding the susceptibility and epidemiology of important respiratory tract pathogens such as *S. aureus*, *H. influenza,* and GABHS [37]. Our results indicates a significant resistance (10.87%) to fusidic acid among *S. aureus* isolates which was similar to Brown and Thomas where they have reported a 10.6% resistance to fusidic acid among methicillin-susceptible *S. aureus* isolates making it a less potential drug of choice for patients with chronic and recurrent tonsillitis [46]. This was also similar to another study by Norazah et al. in which they reported an increased resistant to fusidic acid between 3 and 5% among Malaysian hospitals [47]. We have found that 12 (2.58%) isolates of *H. influenzae* were *β*-lactamase negative ampicillin-resistant (BLNAR). This is of clinical significance, since *H. influenzae* isolates that are BLNAR are typically coresistant to other commonly prescribed *β*-lactams including cephalosporins, amoxicillin-clavulanate, and ampicillin-sulbactam [35].

The recovered ESKAPE pathogens showed high susceptibility against the selected antibiotics except for *P. aeruginosa* where it exhibited 22% resistance to amikacin, ampicillin-sulbactam, amoxicillin-clavulanic acid and ampicillin. This was in contrast with Rice where they reported high levels of resistance by ESKAPE isolates [48]. Bacterial interference has been shown to exist between isolates of *α*-haemolytic and *β*-haemolytic streptococci and between Gram-negative bacilli and *α*-haemolytic streptococci [49]. The lack of interference strains may explain the increased susceptibility of certain individuals to *β*-haemolytic streptococci. Since the administration of antimicrobial agents can affect the composition of the nasopharyngeal flora, a proper use of antibiotics is important in the preservation of the normal interfering flora [50].

The lack of a rapid and reproducible assay to provide a measurable antimicrobial activity against sessile bacteria represents a problem in the selection of alternative antibiotic regiments [51, 52]. Therefore, it is believed that clinical microbiology laboratories can adapt alternative diagnostic techniques to assess the susceptibility of bacteria in the biofilm. This will assist clinicians in the selection of more powerful antibiotics for their activity and efficacy.

The mean age of patients with tonsillitis where streptococci was mainly recovered, that is, streptococcal tonsillitis (ST), was 10 years old while in Nonstreptococcal tonsillitis (NST) it was 13.34 years old. The prevalence of patients with ST was significantly less than that in patients with NST which emphasizes the role of group B, C, G, and F in the clinical presentation and pathogenesis of tonsillitis infections. Our results indicate that the prevalence of bacterial biofilms among ST cases was 100% while among NST cases it was 53% indicating a role of both GABHS and non-GABHS isolates in the pathogenesis of biofilm-associated tonsillar diseases. This was similar to Diaz et al. [9] in which they have reported the correlation between chronic inflammation of the tonsils, clinical features, and the presence of biofilms among 36 patients undergoing tonsillectomy for obstructive sleep apnea and recurrent upper airway infection.

The gross pathology of tonsillar specimens showed that the highest rate of tonsillar grading was grade III (55.71%) followed by grade II (28.57%), grade I (8.57%) and grade IV (7.15%). This was similar to Dell'Aringa et al. (2005) in which they have reported grade III to be the highest with 160 (64%) patients followed by grade II with 45 (18%) patients, grade IV with 26 (10.4%) patients, and grade I with 9 (3.6%) patients [53]. In our study, there was no malignant neoplasia among our tonsillar specimens which can be attributed to the low prevalence of adult patients submitted to tonsillectomy. Moreover, there was no evaluation of the influence of tonsillar size on patients with obstructive sleep apnea and the influence of oropharyngeal anatomy and body mass index on the actual volume of tonsils; this was mainly because we have emphasized more on the microbiology, histopathology, and clinical aspects of tonsillar diseases rather than the physiological and anatomic aspects; therefore, no such correlation was assessed.

Based on the histopathology examination, only 11 (15.71%) patients presented infections by *Actinomyces* spp. in their tonsils leading to tonsillar hypertrophy and an inflammatory lesion of the crypts. This was similar to Pransky et al. where they have reported the presence actinomycosis in 8.5% of patients with obstructive tonsillar hypertrophy and recurrent tonsillitis [54]. However, it was in contrast to Dell'Aringa et al. where they have reported only 2 patients (0.8%) with *Actinomyces *spp. infections [53]. Despite the presence of *Actinomyces* in our examined tonsils, the rate of these infections was significantly low (*P* < 0.05) suggesting no relation between the presence of *Actinomyces *and the hypertrophy of tonsils. This was mainly due to the fact that tonsillar surface is contaminated with oropharyngeal secretions which generally shows normal flora of the oropharynx such as *α*-hemolytic and nonhemolytic streptococci, coagulase negative staphylococci, *Neisseria*, *Corynebacterium*, *Actinomyces*, *Leptotrichia*, and *Fusobacterium* spp. [55].

In our study, bacterial biofilms were present in 60% of tonsils. This was similar to previous findings were biofilms were present in 61% and 70% of examined tonsils, respectively [6, 18]. The high prevalence of biofilm among our tonsils suggests that chronic and recurrent tonsillitis and obstructive sleep apnea are caused by biofilm-forming pathogens. This was similar to previous investigators where they have confirmed the hypothesis that chronic and recurrent tonsillitis are biofilm-related [56, 57]. Although we were able to capture images from different tonsillar specimens to minimize any potential error, these might not completely prevent the chance for false positive biofilm-like artifacts. Therefore, detecting the bacteria and its glycocalyx is crucial for a fundamental understanding of the presence of biofilms in clinical specimens. The use of concanavalin A that binds to mannose residues specific to the bacteria's glycocalyx coupled with CLSM has enabled us to visualize the biofilm's structure more clearly which was similar to a previous study [58]. However, despite the importance of CLSM as a versatile tool it has the limitation of identifying the type of microorganism(s) causing that biofilm in addition of being costly. Furthermore, we could not assess the role of fungi and viruses among our samples due to the technical difficulties in collecting, transporting, and culturing them. Therefore, further studies are needed to tackle the role of nonbacterial biofilms among larger sample size for a better and more insightful understanding of biofilm-associated infections. It has been reported that hypertrophied tonsils even without a history of infection cannot be considered as control samples which is considered as a limitation to our study due to difficulties in obtaining tonsillar specimens from age-matched individuals who never had infection or obstruction in their upper airways. This is similar to the study by Stewart and Costerton where they reported [59]. The evidence of biofilms in the tonsils of patients without a clinical history of infection does raise the possibility that biofilm formation within the tonsillar crypt is part of an immunological surveillance process which leads us to conclude that bacterial biofilms are part of the tonsillar microbial flora among clinically diseased tonsils [18]. Another explanation is that tonsils of healthy individuals are colonized with the same biofilm-forming strains found in tonsillitis patients. However, these strains might not induce a disease. Future studies can be addressed to identify a control group that comprises of volunteers scheduled for laryngeal microsurgery with no history of tonsillitis over the previous 2 years [60].

Our results investigated the correlation between tonsillar inflammations, clinical features, and the presence of biofilms among patients with recurrent infections and obstructive hypertrophy suggesting that biofilm acts a reservoir to establish a persistent infection that leads to the enlargement of tonsils. This was similar to Diaz et al. [9] in which they have demonstrated the symptoms like harsh raucous sound, tonsillar and adenoids hypertrophy, apnea, and cervical adenopathies to be related to the presence of biofilm in the tonsils. Despite the low prevalence of symptoms like apnea and nasal obstruction in comparison with tonsillar and adenoid hypertrophy due to small sample size, a direct correlation between apnea and nasal obstruction was found with the presence of biofilms in 7 out of 12 tonsils within the obstructive group. Moreover, biofilms were found in all hypertrophic tonsils which confirms that tonsillar hypertrophy is one of the important symptoms associated with the presence of biofilms. The increased number of tonsillar lymphatic follicles was related to the presence of biofilms in infected more than hypertrophied tonsils; this finding was similar to a finding in a previous study [61, 62]. The biofilm as a structure is too big to be engulfed by the host's macrophages; therefore their presence in the tonsils will interfere with the normal functions of tonsillar lymphatic tissue which eventually leads to establish a chronic or recurrent infection [62, 63]. This process explains the poor outcome of most therapeutic strategies to minimize the enlarged size of tonsils and avoid the choice of surgery [64]. Failure to respond to antimicrobial therapy leaves the tonsillitis patients with no choice but surgery. However, despite the role of tonsillectomy in relieving the symptoms of tonsillar diseases, the more likely explanation for its effectiveness is the elimination of a possible biofilm infection.

In conclusion, evidence of bacterial biofilms in the tonsils in correlation with the demonstrated clinical symptoms explains the recalcitrant nature of chronic and recurrent tonsillitis and highlights the importance of investigating the microbiology and histopathology of tonsillar diseases towards better therapeutic management of biofilm-related infections.

## Figures and Tables

**Table 1:** Association between the clinical symptoms of tonsillar diseases and the presence of biofilms in tonsils.

Clinical symptom	Patients with clinical symptom	Patients with evidence of biofilm
(1) Tonsillar hypertrophy	49 (70%)*	42 (60%)*
(2) Sore throat	40 (57%)*	40 (57%)*
(3) Adenoid hypertrophy	26 (37.14%)*	13 (18%)*
(4) Apnea	12 (17.14%)*	10 (14%)*
(5) Nasal obstruction	12 (17.14%)*	10 (14%)*

*Percentage was calculated based on the total number of patients which was 70.

**Table 2:** Distribution of bacterial isolates among tonsillar specimens.

Gram-positive isolates	Tonsillar biopsy (core) no. (%)	Tonsillar swab (surface) no. (%)	Total no. (%)
*Staphylococcus aureus *	85 (18.31%)	99 (21.33%)	184 (39.65%)
*Streptococcus agalactiae *	36 (7.75%)	20 (4.31%)	56 (12.06%)
Group G streptococci	11 (2.37%)	14 (3.01%)	25 (5.38%)
*Streptococcus pyogenes *	6 (1.29%)	8 (1.72%)	14 (3.01%)
Group F streptococci	5 (1.07%)	6 (1.29%)	11 (2.37%)
Group C streptococci	4 (0.86%)	4 (0.86%)	8 (1.72%)
*Streptococcus pneumoniae *	1 (0.21%)	2 (0.43%)	3 (0.64%)
Methicillin resistant* S. aureus *	0	1 (0.21%)	1 (0.21%)
Subtotal	**148 (31.89%)**	**154 (33.18%)**	**302 (65.08%)**

Gram-negative isolates	Tonsillar biopsy (core) no. (%)	Tonsillar swab (surface) no. (%)	Total no. (%)

*Haemophilus influenzae *	44 (9.48%)	42 (9.05%)	86 (18.53%)
*Haemophilus parainfluenzae *	10 (2.15%)	21 (4.52%)	31 (6.68%)
*Klebsiella pneumoniae *	15 (3.23%)	15 (3.23%)	30 (6.46%)
*Pseudomonas aeruginosa *	5 (1.07%)	4 (0.86%)	9 (1.93%)
*Citrobacter *sp.	2 (0.43%)	2 (0.43%)	4 (0.86%)
*Acinetobacter baumannii *	1 (0.21%)	0	1 (0.21%)
*Enterobacter cloacae *	0	1 (0.21%)	1 (0.21%)
Subtotal	**77 (16.59%)**	**85 (18.31%)**	**162 (34.91%)**
Total	**225 (48.49%)**	**239 (51.50%)**	**464 (100%)**
